# Longitudinal study of diabetes prevalence and hospitalisations among care experienced and general population children in Scotland: evidence of an end of care “cliff edge”?

**DOI:** 10.23889/ijpds.v7i3.1998

**Published:** 2022-08-25

**Authors:** Mirjam Allik, Alastair Leyland, Marion Henderson

**Affiliations:** 1 University of Glasgow; 2 University of Strathclyde

## Objectives

Care experienced people have poorer health in UK and internationally, but the direction of causation is debated. Using longitudinal cross-sectoral data linkage we explore if inequalities in diabetes prevalence and hospitalisation are present before entering care or develop during or after leaving care.

## Approach

Health and social care data were linked for 13,830 care experienced children (CEC) and together with 649,771 general population children (GPC) their prescriptions and hospitalisations were followed from birth between 1990-2004 to study end in 2016. Diabetes prevalence was estimated as at least one prescription or inpatient hospitalisation for diabetes. We compared hospitalisation percentages and rates in the two cohorts by age and gender. Results from multivariable models adjusted for socioeconomic status, age, gender, care type/length, local authority, and comorbidities will be presented at conference.

## Results

Diabetes prevalence was similar in both cohorts and higher in females. However, CEC had twice as many hospitalisations as GPC. Mean hospitalisations were highest among care experienced males (6 compared to 3.6 in females and 2 in GPC). 24% of CEC were hospitalised 3-9 times and 13% 10+ times, for GPC these were 19% and 3% respectively. Hospitalisation rates increase with age in both cohorts, as do differences between cohorts. At ages 0-4 hospitalisation rates are similar, by ages 12-15 CEC have twice as high and at ages 18-27 4-times higher hospitalisation rates. Among CEC, across all ages hospitalisation rates are lower while the child is in care, with the lowest rates in foster care. Hospitalisation rates are highest before entering and after leaving care.

## Conclusion

Results for diabetes hospitalisations suggest that being in care can be good for children’s health. However, a sudden withdrawal of support can create a “cliff edge” and health may deteriorate after leaving care. Data linkage has significant potential to inform policy and practice, including supporting CEC after leaving care.

